# Supplementary material for: Identification of Freezing-Responsive microRNAs and Their Targets in Chinese Jujube by Small RNA and Degradome Sequencing
Source: Int J Mol Sci. 2026 Sep 4;27(17):7912. doi: 10.3390/ijms27177912 (PMC13566890; doi:10.3390/ijms27177912)
Supplement: Supplementary file 1 [file ijms-27-07912-s001.zip › Supplementary Material S2/novel_miR_12.pdf]

Provisional ID	:	novel_miR_12
Score total	:	3.1
Score for star read(s)	:	-1.3
Score for read counts	:	0
Score for mfe	:	2.8
Score for randfold	:	1.6
Score for cons. seed	:	
Total read count	:	1295
Mature read count	:	1067
Loop read count	:	228
Star read count	:	0

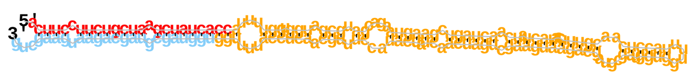

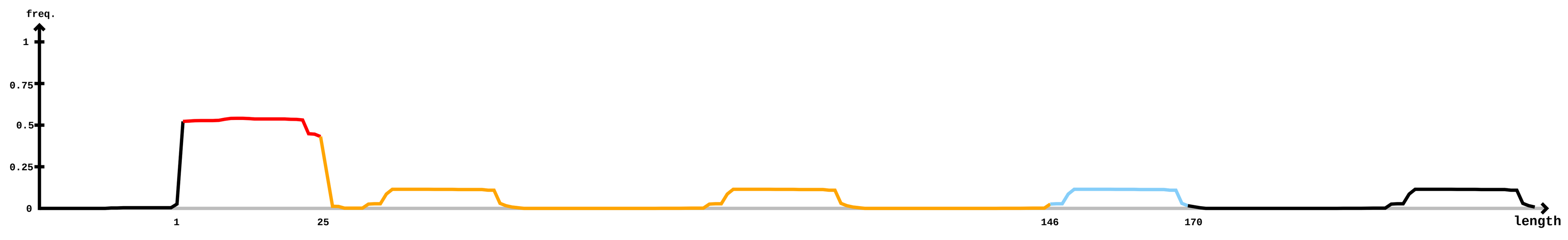


```
                     Mature                                                                                                                       Star
5'- uaacagguguaaugguugacuaacuuccuucugcuaagcuaucaccauuuuggugguagcguagcagaaugaagcugaucaacuaucauaccuuugcaacuccauuuuggugguagcguagcagaaugaagcugaucaacuaucauaccuuugcaacuccauuuuggugguagcguagcagaaugaagcugaucaacuaucauaccuuugcaacuccauuuuggugguagcguagcagaaugaagcugau -3'  exp
    ....((((((.((((((((.((.((((.((((((((.(((((((((...(((.(((.(((.((....((((((.((((((.((.((((...(((((..((((((....)))).))....))))))))))).)))))).)).))))..)).))).))))))...)))))))))).))))))))).)))).)).)))))))).))))))...((.((.(((((((.((......)).))))))).)).))..  reads  mm  sample
    ..........aauggCugacuaacuuccuucugc........................................................................................................................................................................................................................  3  1  JCK-2
    ..........aauggCugacuaacuuccuucugc........................................................................................................................................................................................................................  1  1  JCK-1
    ..........aauggCugacuaacuuccuucugc........................................................................................................................................................................................................................  1  1  JCK-3
    ............uggCugacuaacuuccuucug.........................................................................................................................................................................................................................  1  1  JCK-1
    ............uggCugacuaacuuccuucug.........................................................................................................................................................................................................................  2  1  JCK-2
    .....................aacuuccuucugcuaagcu..................................................................................................................................................................................................................  1  0  JCK-2
    .....................aacuuccuucugcuaagcu..................................................................................................................................................................................................................  1  0  JCK-1
    .....................aacuuccuucugcuaagcua.................................................................................................................................................................................................................  1  0  LCK-3
    .....................aacuuccuucugcuaagcuUu................................................................................................................................................................................................................  1  1  LT-1
    .....................aacuuccuucugcuaagcuau................................................................................................................................................................................................................  1  0  JCK-3
    .....................aacuuccuucugcuaagcuau................................................................................................................................................................................................................  4  0  JCK-2
    .....................aacuuccuucugcuaagcuau................................................................................................................................................................................................................  1  0  LT-3
    .....................aacuuccuucugcuaagcuauc...............................................................................................................................................................................................................  2  0  JCK-3
    .....................aacuuccuucugcuaagcuauc...............................................................................................................................................................................................................  3  0  JCK-2
    .....................aacuuccuucugcuaagcuauc...............................................................................................................................................................................................................  3  0  JCK-1
    .....................aacuuccuucugcuaagcuauca..............................................................................................................................................................................................................  1  0  JCK-1
    .....................aacuuccuucugcuaagcuauca..............................................................................................................................................................................................................  1  0  JCK-2
    .....................aacuuccuucugcuaagcuaucac.............................................................................................................................................................................................................  7  0  JCK-1
    .....................aacuuccuucugcuaagcuaucac.............................................................................................................................................................................................................  6  0  JCK-2
    .....................aacuuccuucugcuaagcuaucac.............................................................................................................................................................................................................  11  0  JCK-3
    ......................acuuccuucugcuaagcA..................................................................................................................................................................................................................  2  1  LT-3
    ......................acuuccuucugcuaagcuauc...............................................................................................................................................................................................................  17  0  LT-1
    ......................acuuccuucugcuaagcuauc...............................................................................................................................................................................................................  35  0  LT-3
    ......................acuuccuucugcuaagcuauc...............................................................................................................................................................................................................  37  0  JCK-2
    ......................acuuccuucugcuaagcuauc...............................................................................................................................................................................................................  6  0  LT-2
    ......................acuuccuucugcuaagcuauc...............................................................................................................................................................................................................  15  0  JCK-1
    ......................acuuccuucugcuaagcuauc...............................................................................................................................................................................................................  25  0  LCK-3
    ......................acuuccuucugcuaagcuauc...............................................................................................................................................................................................................  21  0  JCK-3
    ......................acuuccuucugcuaagcuauca..............................................................................................................................................................................................................  2  0  JCK-3
    ......................acuuccuucugcuaagcuauca..............................................................................................................................................................................................................  1  0  LT-3
    ......................acuuccuucugcuaagcuaucac.............................................................................................................................................................................................................  1  0  JCK-2
    ......................acuuccuucugcuaagcuaucac.............................................................................................................................................................................................................  2  0  JCK-3
    ......................acuuccuucugcuaagcuaucac.............................................................................................................................................................................................................  1  0  JCK-1
    ......................acuuccuucugcuaCgcuaucacc............................................................................................................................................................................................................  1  1  JCK-2
```

Mature Star

```
uaacagguguaaugguugacuaacuuccuucugcuaagcuaucaccauuuuggugguagcguagcagaaugaagcugaucaacuaucauaccuuugcaacuccauuuuggugguagcguagcagaaugaagcugaucaacuaucauaccuuugcaacuccauuuuggugguagcguagcagaaugaagcugaucaacuaucauaccuuugcaacuccauuuuggugguagcguagcagaaugaagcugau
......................acuuccuucugcuaagcuaucacc............................................................................................................................................................................................  21   0  LT-3
......................acuNccuucugcuaagcuaucacc............................................................................................................................................................................................  1    1  JCK-1
......................acuuccuucugcuaagcuaucacc............................................................................................................................................................................................  160  0  JCK-1
......................acuucUuucugcuaagcuaucacc............................................................................................................................................................................................  1    1  JCK-3
......................acuucUuucugcuaagcuaucacc............................................................................................................................................................................................  2    1  JCK-1
......................acuuccuucugcuaagcuaucacc............................................................................................................................................................................................  9    0  LT-1
......................acuuccuucugcuaagcuaucacc............................................................................................................................................................................................  7    0  JT-2
......................acuuccuucugcuaagcuaCcacc............................................................................................................................................................................................  1    1  JCK-1
......................acuuccuucugcuaagcuaucaAc............................................................................................................................................................................................  1    1  JCK-2
......................acuuccuucugcuaagcuaucacc............................................................................................................................................................................................  7    0  JT-3
......................acuuccuucugcuaagcuaucacc............................................................................................................................................................................................  22   0  LT-2
......................acuuccuucugcuaagcuCucacc............................................................................................................................................................................................  1    1  LT-3
......................acuuccuucugcuaagcuaucacA............................................................................................................................................................................................  1    1  JCK-2
......................acuuccuucugcuCagcuaucacc............................................................................................................................................................................................  1    1  JCK-2
......................acuuccuucugcuaagcuaucacc............................................................................................................................................................................................  57   0  LCK-2
......................acuuccuucugcuaagcuaucacc............................................................................................................................................................................................  262  0  JCK-2
......................acuuccuucugcuaagcuGucacc............................................................................................................................................................................................  1    1  JCK-2
......................aNuuccuucugcuaagcuaucacc............................................................................................................................................................................................  1    1  JCK-3
......................acuuccuucugcuaagcuaucacA............................................................................................................................................................................................  1    1  LT-1
......................acuuccuucugcuCagcuaucacc............................................................................................................................................................................................  1    1  LCK-1
......................acCuccuucugcuaagcuaucacc............................................................................................................................................................................................  1    1  JCK-3
......................acuucUuucugcuaagcuaucacc............................................................................................................................................................................................  2    1  JCK-2
......................acuuccuucugcuaagcuaucacc............................................................................................................................................................................................  189  0  JCK-3
......................acuuccuucugcuaagcuaucacc............................................................................................................................................................................................  41   0  LCK-1
......................aUuuccuucugcuaagcuaucacc............................................................................................................................................................................................  1    1  JCK-3
......................acuuccuucugcuaagcuaucacca...........................................................................................................................................................................................  7    0  JCK-2
......................aNuuccuucugcuaagcuaucacca...........................................................................................................................................................................................  1    1  JCK-2
......................acuuccuucugcuaagcuaucacca...........................................................................................................................................................................................  4    0  LCK-3
......................acuuccuucugcuaagcuaucacca...........................................................................................................................................................................................  11   0  JCK-3
......................acuuccuucugcuaagcuaucacca...........................................................................................................................................................................................  2    0  LT-1
......................acuuccuucugcuaagcuaucacca...........................................................................................................................................................................................  3    0  JCK-1
.......................cuuccuucugcuaagcuaucac.............................................................................................................................................................................................  1    0  JCK-2
.......................cuuccuucugcuaagcuaucac.............................................................................................................................................................................................  1    0  JCK-3
.......................cuuccuucugcuaagcuaucacc............................................................................................................................................................................................  2    0  JCK-3
........................uuccuucugcuaagcuaucacc............................................................................................................................................................................................  2    0  JCK-3
........................uuccuucugcuaagcuaucacc............................................................................................................................................................................................  3    0  JCK-2
.........................uccuucugcuaagcuaucacc............................................................................................................................................................................................  1    0  JCK-2
............................uucugcuaagcuaucacca...........................................................................................................................................................................................  1    0  JCK-2
............................uucugcuaagcuaucaccauu.........................................................................................................................................................................................  2    0  LT-1
.............................ucugcuaagcuaucacca...........................................................................................................................................................................................  2    0  JCK-1
.............................Ccugcuaagcuaucaccauu.........................................................................................................................................................................................  11   1  LCK-3
..............................cugcuaagcuaucaccau..........................................................................................................................................................................................  1    0  LT-3
..............................cugcuaagcuaucaccau..........................................................................................................................................................................................  1    0  LT-1
..............................cugcuaagcuaucaccauu.........................................................................................................................................................................................  4    0  JCK-2
..............................cugcuaagcuaucaccauu.........................................................................................................................................................................................  3    0  LT-3
..............................cugcuaagcuaucaccauu.........................................................................................................................................................................................  1    0  LT-2
...............................ugcuaagcuaucaccauuu........................................................................................................................................................................................  1    0  LT-3
.............................................cauuuuggugguagcguag..........................................................................................................................................................................  4    0  JCK-2
.................................................uuggugguagcguagcaga.......................................................................................................................................................................  4    0  LT-3
..................................................uggugguagcguagcaga.......................................................................................................................................................................  4    0  JCK-1
.....................................................ugguagcguagcagaaugaa.................................................................................................................................................................  12   0  JT-2
.....................................................ugguagcguagcagaaugaa.................................................................................................................................................................  20   0  JT-3
.....................................................ugguagcguagcagaaugaagc...............................................................................................................................................................  28   0  JCK-2
.....................................................ugguagcguagcagaaugaagc...............................................................................................................................................................  28   0  JCK-3
.....................................................ugguagcguagcagaaugaagc...............................................................................................................................................................  36   0  JCK-1
.....................................................ugguagcguagcagaaugaagc...............................................................................................................................................................  4    0  LT-1
.....................................................ugguagcguagcagaaugaagcC..............................................................................................................................................................  4    1  JCK-2
.....................................................ugguagcguagcagaaugaagcug.............................................................................................................................................................  4    0  JCK-1
.....................................................ugguagcguagcagaaugaagcug.............................................................................................................................................................  20   0  JCK-2
.....................................................ugguagcguagcagaaugaagcug.............................................................................................................................................................  4    0  LT-3
.....................................................ugguagcguagNagaaugaagcug.............................................................................................................................................................  4    1  JCK-1
.....................................................ugguagcguagcagaaugaagcuga............................................................................................................................................................  4    0  JCK-2
.....................................................ugguagcguagcagaaugaagcugaA...........................................................................................................................................................  12   1  JCK-2
.....................................................ugguagcguagcagaaugaagcugau...........................................................................................................................................................  8    0  LT-2
.....................................................ugguagcguagcagaaugaagcugaA...........................................................................................................................................................  4    1  JCK-3
......................................................gguagcguagcagaaugaagcug.............................................................................................................................................................  4    0  JCK-2
......................................................gguagcguagcagaaugaagcuga............................................................................................................................................................  8    0  JCK-2
......................................................gguagcguagcagaaugaagcuga............................................................................................................................................................  4    0  JCK-1
........................................................uagcguagcagaaugaagU...............................................................................................................................................................  4    1  LT-3
........................................................uagcguagcagaaugaagc...............................................................................................................................................................  148  0  LCK-3
```

Mature · **Star**

uaacagguguaaugguugacuaacuuccuucugcuaagcuaucaccauuuugguggguagcguagcagaaugaagcugaucaacuaucauaccuuugcaacuccauuuugguggguagcguagcagaaugaagcugaucaacuaucauaccuuugcaacuccauuuuggugguagcguagcagaaugaagcugaucaacuaucauaccuuugcaacuccauuuugguggguagcguagcagaaugaagcugau

uagcguagcagaaugaagc	8	0	JCK-3
uagcguagcagaaugaagc	56	0	LT-2
uagcguagcagaaugaagc	56	0	LT-1
uagcguagcagaaugaagc	40	0	LT-3
uagcguagcagaaugaaUc	12	1	LT-3
uagcguagcagaaugaagUu	4	1	JCK-1
uagcguagcagaaugaagUu	12	1	LT-2
uagcguagcagaaugaagUu	52	1	LCK-3
uagcguagcagaaugaagUu	4	1	JCK-2
uagcguagcagaaugaagUu	20	1	LT-3
uagcguagcagaaugaagUu	12	1	JCK-3
uagcguagcagaaugaagcug	4	0	JCK-1
uagcguagcagaaugaagcug	12	0	LT-1
uagcguagcagaaugaagcug	4	0	LT-3
uagcguagcagaaugaagcug	8	0	JCK-2
uagcguagcagaaugaagcugau	4	0	JCK-3
uagcguagcagaaugaagcugau	4	0	JCK-2
agcguagcagaaugaagc	36	0	LT-3
agcguagcagaaugaagc	4	0	JT-3
agcguagcagaaugaagc	8	0	JT-2
agcguagcagaaugaagc	16	0	JCK-2
agNguagcagaaugaagc	4	1	LT-2
agcguagcagaaugaagc	28	0	JCK-3
agcguagcagaaugaagc	40	0	JCK-1
agcguagcagaaugaagc	12	0	LT-1
agcguagcagaaugaagc	60	0	LT-2
agcguagcagaaugaagcC	4	1	LT-1
agcguagcagaaugaagcuga	8	0	JCK-3
agcguagcagaaugaagcuga	8	0	JCK-2
cauuuuggugguagcguag	4	0	JCK-2
uuggugguagcguagcaga	4	0	LT-3
uggugguagcguagcaga	4	0	JCK-1
ugguagcguagcagaaugaa	12	0	JT-2
ugguagcguagcagaaugaa	20	0	JT-3
ugguagcguagcagaaugaagc	28	0	JCK-2
ugguagcguagcagaaugaagc	36	0	JCK-1
ugguagcguagcagaaugaagc	4	0	LT-1
ugguagcguagcagaaugaagc	28	0	JCK-3
ugguagcguagcagaaugaagcC	4	1	JCK-2
ugguagcguagcagaaugaagcug	4	0	LT-3
ugguagcguagNagaaugaagcug	4	1	JCK-1
ugguagcguagcagaaugaagcug	20	0	JCK-2
ugguagcguagcagaaugaagcug	4	0	JCK-1
ugguagcguagcagaaugaagcuga	4	0	JCK-2
ugguagcguagcagaaugaagcugaA	4	1	JCK-3
ugguagcguagcagaaugaagcugaA	12	1	JCK-2
ugguagcguagcagaaugaagcugau	8	0	LT-2
gguagcguagcagaaugaagcug	4	0	JCK-2
gguagcguagcagaaugaagcuga	4	0	JCK-1
gguagcguagcagaaugaagcuga	8	0	JCK-2
uagcguagcagaaugaagc	148	0	LCK-3
uagcguagcagaaugaagc	56	0	LT-1
uagcguagcagaaugaagU	4	1	LT-3
uagcguagcagaaugaagc	8	0	JCK-3
uagcguagcagaaugaaUc	12	1	LT-3
uagcguagcagaaugaagc	56	0	LT-2
uagcguagcagaaugaagc	40	0	LT-3
uagcguagcagaaugaagUu	4	1	JCK-2
uagcguagcagaaugaagUu	20	1	LT-3
uagcguagcagaaugaagUu	4	1	JCK-1
uagcguagcagaaugaagUu	12	1	JCK-3
uagcguagcagaaugaagUu	52	1	LCK-3
uagcguagcagaaugaagUu	12	1	LT-2
uagcguagcagaaugaagcug	4	0	LT-3
uagcguagcagaaugaagcug	8	0	JCK-2
uagcguagcagaaugaagcug	12	0	LT-1
uagcguagcagaaugaagcug	4	0	JCK-1
uagcguagcagaaugaagcugau	4	0	JCK-2
uagcguagcagaaugaagcugau	4	0	JCK-3
agcguagcagaaugaagc	4	0	JT-3

Mature Star

uaacagguguaaugguugacuaacuuccuucugcuaagcuaucaccauuuuggugguagcguagcagaaugaagcugaucaacuaucauaccuuugcaacuccauuuuggugguagcguagcagaaugaagcugaucaacuaucauaccuuugcaacuccauuuuggugguagcguagcagaaugaagcugaucaacuaucauaccuuugcaacuccauuuuggugguagcguagcagaaugaagcugau

agcguagcagaaugaagc	40	0	JCK-1
agcguagcagaaugaagc	28	0	JCK-3
agcguagcagaaugaagc	12	0	LT-1
agcguagcagaaugaagc	60	0	LT-2
agcguagcagaaugaagc	16	0	JCK-2
agcguagcagaaugaagc	36	0	LT-3
agcguagcagaaugaagc	8	0	JT-2
agNguagcagaaugaagc	4	1	LT-2
agcguagcagaaugaagcC	4	1	LT-1
agcguagcagaaugaagcuga	8	0	JCK-2
agcguagcagaaugaagcuga	8	0	JCK-3
cauuuuggugguagcguag	4	0	JCK-2
uuggugguagcguagcaga	4	0	LT-3
uggugguagcguagcaga	4	0	JCK-1
ugguagcguagcagaaugaa	20	0	JT-3
ugguagcguagcagaaugaa	12	0	JT-2
ugguagcguagcagaaugaagc	28	0	JCK-3
ugguagcguagcagaaugaagc	4	0	LT-1
ugguagcguagcagaaugaagc	28	0	JCK-2
ugguagcguagcagaaugaagc	36	0	JCK-1
ugguagcguagcagaaugaagcC	4	1	JCK-2
ugguagcguagcagaaugaagcug	4	0	LT-3
ugguagcguagcagaaugaagcug	20	0	JCK-2
ugguagcguagNagaaugaagcug	4	1	JCK-1
ugguagcguagcagaaugaagcug	4	0	JCK-1
ugguagcguagcagaaugaagcuga	4	0	JCK-2
ugguagcguagcagaaugaagcugaA	4	1	JCK-3
ugguagcguagcagaaugaagcugau	8	0	LT-2
ugguagcguagcagaaugaagcugaA	12	1	JCK-2
gguagcguagcagaaugaagcug	4	0	JCK-2
gguagcguagcagaaugaagcuga	8	0	JCK-2
gguagcguagcagaaugaagcuga	4	0	JCK-1
uagcguagcagaaugaagc	8	0	JCK-3
uagcguagcagaaugaaUc	12	1	LT-3
uagcguagcagaaugaagc	56	0	LT-1
uagcguagcagaaugaagU	4	1	LT-3
uagcguagcagaaugaagc	148	0	LCK-3
uagcguagcagaaugaagc	40	0	LT-3
uagcguagcagaaugaagc	56	0	LT-2
uagcguagcagaaugaagUu	12	1	JCK-3
uagcguagcagaaugaagUu	4	1	JCK-1
uagcguagcagaaugaagUu	20	1	LT-3
uagcguagcagaaugaagUu	4	1	JCK-2
uagcguagcagaaugaagUu	52	1	LCK-3
uagcguagcagaaugaagUu	12	1	LT-2
uagcguagcagaaugaagcug	12	0	LT-1
uagcguagcagaaugaagcug	4	0	LT-3
uagcguagcagaaugaagcug	8	0	JCK-2
uagcguagcagaaugaagcug	4	0	JCK-1
uagcguagcagaaugaagcugau	4	0	JCK-2
uagcguagcagaaugaagcugau	4	0	JCK-3
agcguagcagaaugaagc	40	0	JCK-1
agcguagcagaaugaagc	36	0	LT-3
agcguagcagaaugaagc	8	0	JT-2
agcguagcagaaugaagc	12	0	LT-1
agNguagcagaaugaagc	4	1	LT-2
agcguagcagaaugaagc	60	0	LT-2
agcguagcagaaugaagc	16	0	JCK-2
agcguagcagaaugaagc	28	0	JCK-3
agcguagcagaaugaagc	4	0	JT-3
agcguagcagaaugaagcC	4	1	LT-1
agcguagcagaaugaagcuga	8	0	JCK-2
agcguagcagaaugaagcuga	8	0	JCK-3
cauuuuggugguagcguag	4	0	JCK-2
uuggugguagcguagcaga	4	0	LT-3
uggugguagcguagcaga	4	0	JCK-1
ugguagcguagcagaaugaa	12	0	JT-2
ugguagcguagcagaaugaa	20	0	JT-3
ugguagcguagcagaaugaagc	28	0	JCK-3
ugguagcguagcagaaugaagc	28	0	JCK-2

Mature **Star**

```
uaacagguguaaugguugacuaacuuccuucugcuaagcuaucaccauuuuggugguagcguagcagaaugaagcugaucaacuaucauaccuuugcaacuccauuuuggugguagcguagcagaaugaagcugaucaacuaucauaccuuugcaacuccauuuuggugguagcguagcagaaugaagcugaucaacuaucauaccuuugcaacuccauuuuggugguagcguagcagaaugaagcugau
................................................................................................................................................................................................................................ugguagcguagcagaaugaagc....   36   0   JCK-1
................................................................................................................................................................................................................................ugguagcguagcagaaugaagc....   4    0   LT-1
................................................................................................................................................................................................................................ugguagcguagcagaaugaagcC...   4    1   JCK-2
................................................................................................................................................................................................................................ugguagcguagcagaaugaagcug..   20   0   JCK-2
................................................................................................................................................................................................................................ugguagcguagNagaaugaagcug..   4    1   JCK-1
................................................................................................................................................................................................................................ugguagcguagcagaaugaagcug..   4    0   LT-3
................................................................................................................................................................................................................................ugguagcguagcagaaugaagcug..   4    0   JCK-1
................................................................................................................................................................................................................................ugguagcguagcagaaugaagcuga.   4    0   JCK-2
................................................................................................................................................................................................................................ugguagcguagcagaaugaagcugau   8    0   LT-2
................................................................................................................................................................................................................................ugguagcguagcagaaugaagcugaA   12   1   JCK-2
................................................................................................................................................................................................................................ugguagcguagcagaaugaagcugaA   4    1   JCK-3
.................................................................................................................................................................................................................................gguagcguagcagaaugaagcug..   4    0   JCK-2
.................................................................................................................................................................................................................................gguagcguagcagaaugaagcuga.   8    0   JCK-2
.................................................................................................................................................................................................................................gguagcguagcagaaugaagcuga.   4    0   JCK-1
..................................................................................................................................................................................................................................uagcguagcagaaugaagc....   40   0   LT-3
..................................................................................................................................................................................................................................uagcguagcagaaugaagc....   56   0   LT-2
..................................................................................................................................................................................................................................uagcguagcagaaugaagc....   8    0   JCK-3
..................................................................................................................................................................................................................................uagcguagcagaaugaaUc....   12   1   LT-3
..................................................................................................................................................................................................................................uagcguagcagaaugaagc....   56   0   LT-1
..................................................................................................................................................................................................................................uagcguagcagaaugaagc....   148  0   LCK-3
..................................................................................................................................................................................................................................uagcguagcagaaugaagU....   4    1   LT-3
..................................................................................................................................................................................................................................uagcguagcagaaugaagUu...   12   1   LT-2
..................................................................................................................................................................................................................................uagcguagcagaaugaagUu...   4    1   JCK-1
..................................................................................................................................................................................................................................uagcguagcagaaugaagUu...   4    1   JCK-2
..................................................................................................................................................................................................................................uagcguagcagaaugaagUu...   20   1   LT-3
..................................................................................................................................................................................................................................uagcguagcagaaugaagUu...   52   1   LCK-3
..................................................................................................................................................................................................................................uagcguagcagaaugaagUu...   12   1   JCK-3
..................................................................................................................................................................................................................................uagcguagcagaaugaagcug..   4    0   LT-3
..................................................................................................................................................................................................................................uagcguagcagaaugaagcug..   4    0   JCK-1
..................................................................................................................................................................................................................................uagcguagcagaaugaagcug..   12   0   LT-1
..................................................................................................................................................................................................................................uagcguagcagaaugaagcug..   8    0   JCK-2
..................................................................................................................................................................................................................................uagcguagcagaaugaagcugau   4    0   JCK-3
..................................................................................................................................................................................................................................uagcguagcagaaugaagcugau   4    0   JCK-2
...................................................................................................................................................................................................................................agcguagcagaaugaagc....   12   0   LT-1
...................................................................................................................................................................................................................................agcguagcagaaugaagc....   4    0   JT-3
...................................................................................................................................................................................................................................agcguagcagaaugaagc....   8    0   JT-2
...................................................................................................................................................................................................................................agcguagcagaaugaagc....   60   0   LT-2
...................................................................................................................................................................................................................................agcguagcagaaugaagc....   40   0   JCK-1
...................................................................................................................................................................................................................................agcguagcagaaugaagc....   36   0   LT-3
...................................................................................................................................................................................................................................agNguagcagaaugaagc....   4    1   LT-2
...................................................................................................................................................................................................................................agcguagcagaaugaagc....   28   0   JCK-3
...................................................................................................................................................................................................................................agcguagcagaaugaagc....   16   0   JCK-2
...................................................................................................................................................................................................................................agcguagcagaaugaagcC...   4    1   LT-1
...................................................................................................................................................................................................................................agcguagcagaaugaagcuga.   8    0   JCK-2
...................................................................................................................................................................................................................................agcguagcagaaugaagcuga.   8    0   JCK-3
```
